# Comparative Chloroplast Genomics and Phylogenetic Analysis of *Thuniopsis* and Closely Related Genera within Coelogyninae (Orchidaceae)

**DOI:** 10.3389/fgene.2022.850201

**Published:** 2022-03-24

**Authors:** Lin Li, Qiuping Wu, Lin Fang, Kunlin Wu, Mingzhi Li, Songjun Zeng

**Affiliations:** ^1^ Guangdong Provincial Key Laboratory of Applied Botany, South China Botanical Garden, Chinese Academy of Sciences, Guangzhou, China; ^2^ University of Chinese Academy of Sciences, Beijing, China; ^3^ Guangzhou Bio and Data Biotechnology Co., Ltd., Guangzhou, China; ^4^ Key Laboratory of South China Agricultural Plant Molecular Analysis and Gene Improvement, South China Botanical Garden, Chinese Academy of Sciences, Guangzhou, China

**Keywords:** *Thunia*, *Thuniopsi*s, chloroplast genome, comparative analysis, phylogeny

## Abstract

The genus *Thuniopsis* was recently proposed for a rare orchid species *T. cleistogama* formerly classified in the genus *Thunia*. The relationships between *Thuniopsis* and its related genera have not yet been conclusively resolved. Recognition of the genus provides a new perspective to illustrate the morphological diversity and plastome evolution within Coelogyninae. In this study, we sequenced and assembled complete chloroplast (cp) genomes for three accessions of *Thuniopsis cleistogama* and two accessions of *Thunia alba.* A total of 135 genes were annotated for each cp genome, including 89 protein-coding genes, 38 tRNA genes, and eight rRNA genes. The ENC-plot and neutrality plot analyses revealed that natural selection dominated over mutation pressure in their evolutionary process. Specially, we found that selection played a vital role in shaping the codon usage in *Thunia alba* cp genome. General characteristics of the cp genomes were further analyzed and compared with those published plastomes of four other related species. Despite the conserved organization and structure, the whole individual cp genome size ranged from 158,394 bp to 159,950 bp. In all the examined plastomes, sequences in the inverted repeat (IR) regions were more conserved than those in the small single copy (SSC) and large single copy (LSC) regions. However, close examination identified contraction and expansion of the IR/SSC boundary regions, which might be the main reason for the cp genome size variation. Our comparative analysis of the cp genomes revealed that single nucleotide polymorphisms (SNPs) and insertions/deletions (InDels) provided valuable information for identifying genetic variations within and among genera. Furthermore, sequence variations in the protein-coding regions were more conserved than those in the non-coding regions. We selected eight divergence hotspots with nucleotide sequence diversities (*Pi* values) higher than 0.08. Most of these polymorphisms were located in the intergenic regions. Phylogenomic analyses recovered largely congruent relationships among major clades and strongly supported the monophyly of *Thuniopsis*. The results obtained in this study can improve our understanding of the classification of this enigmatic genus. The chloroplast genomic data presented here provide valuable insights into the phylogeny and evolutionary patterns of the Coelogyninae as well as the orchids as a whole.

## Introduction

The genus *Thuniopsis* L. Li, D.P.Ye and Shi.J.Li, belonging to subtribe Coelogyninae of tribe Arethuseae (Pridgeon et al., 2005) was recently established to accommodate an unusual species *Thuniopsis cleistogama* from Yunnan, China (Li et al., 2015). As the elongate leafy stem and foliage resemble those of the genus *Thunia* Rchb. f., this species has originally assigned to the member of that genus (Xu et al., 2010). Recently it was considered to be congeneric with a long-lost and rediscovered species in Myanmar formerly classified in the genus *Arundina* or *Dilochia* (Kang et al., 2019; Kurzweil et al., 2020). Owing to remarkable vegetative similarity in morphology, the species has long been mistakenly identified as a member of other genera for over 100 years. In China, it was considered to be a rare and endangered species due to a narrow distribution. Unfortunately, previous phylogenetic study of tribe Arethuseae based on three gene regions (nuclear ribosomal ITS, chloroplast *matK* and *trnL*), although generally well supported among major clades, its phylogenetic relationships of *Thuniopsis* with related genera, remain unclear, as the internal node was not highly resolved (Li et al., 2015).

The chloroplast genomes of plants are highly conserved in sequence and structure due to their maternal inherited and the moderate evolutionary rate (Wicke et al., 2011). Analysis of the complete chloroplast genome sequences has been widely used to infer phylogenetic relationships and provide data useful in molecular evolution (Lu et al., 2017; Lee et al., 2019). In the present study, the cp genome sequences of three individuals of *Thuniopsis cleistogama*, and two individuals of *Thunia alba* were newly assembled and annotated. Codon usage analysis was conducted to find the codon bias. Simple sequence repeats (SSRs) were used to investigate the genetic diversity of these plastomes. Further, polymorphic regions were identified by comparing new sequenced chloroplast genomes with four published genomes of related genera. Based on plastome data, this study aims to reconstruct a robust phylogeny to infer the systematic placement of *Thuniopsis* and elucidate the phylogenetic relationships of the members within subtribe Coelogyninae. The present results provide a useful genetic resource for molecular identification and evolutionary studies of *Thuniopsis* and its related genera.

## Materials and Methods

### Sampling, DNA Extraction and Sequencing

Fresh mature leaves were plucked from two to three plant individuals sampled for *Thunia alba* (L01 and L02) and *Thuniopsis cleistogama* (L03, L04 and L05). The living plants were introduced from Pu’er, Yunnan Province in Southewest China and cultivated in the greenhouse of South China Botanical Garden, Chinese Academy of Science (SCBG, CAS). Total genomic DNA was extracted from young leaves using a Trelief TM Plant Genomic DNA Kit (TsingKe Biotechnology Co., Ltd., Beijing, China). After quality testing, DNA was fragmented and used to set up 200 bp short-insert libraries and the qualified libraries were sequenced with PE150 bp on the BGISEQ-500 sequencer according to the manufacturer’s instructions. The sequencing depth was ∼3.0 Gb of 150-bp paired-end reads for each species. Voucher specimens were deposited at the herbarium of South China Botanical Garden, CAS (IBSC).

### Plastome Assembly and Annotation

First, all raw reads were trimmed using fastp (Chen et al., 2018). Subsequently, high-quality reads were mapped to the reference chloroplast genomes of Orchidaceae obtained from GenBank through Bowtie2 v.2.3.4.3 (Langmead and Salzber, 2012). The sequence of the coding gene having the maximum coverage was utilized as a seed sequence for de-novo assembly by NOVOPlasty v4.2.1 (Dierckxsens et al., 2017). To ensure the accuracy of the genome assembly, raw sequencing reads were remapped to the candidate chloroplast genome. The final assembly included contiguous sequence without gaps. The assembled cp genomes were annotated with GeSeq (Tillich et al., 2017) and tRNAscan (Chan and Lowe, 2019), then manual adjusted and confirmed using Geneious 9.1.8 (Kearse et al., 2012). The circular chloroplast genome map was drawn by OrganellarGenomeDRAW tool (OGDRAW) v.1.3.1 (Greiner et al., 2019) for further comparison of gene order and content.

### Plastome Comparison and Sequence Divergence Analysis

The codon usage patterns of protein-coding genes in the *Thuniopsis cleistogama* genomes (L03, L04 and L05) and *Thunia alba* genomes (L01 and L02) were estimated by the software CodonW v.1.4.2. The Relative Synonymous Codon Usage (RSCU) values and the effective number of codon (ENC) values were determined to quantify the extent of the codon usage bias. RSCU was calculated for every codon in each genome according to the published equation (Sharp and Li, 1987). The ENC values indicate the codon bias for individual genes, over a range of values from 20 to 61. Lower ENC values indicate higher codon bias (Wright et al., 1990). The overall GC content and GC content at the first, second and third codon positions (GC1, GC2, and GC3, respectively) as well as the average GC content of the first and second codon positions (GC12) of the genomes were calculated using EMBOSS software suite (Rice et al., 2000). The neutrality plot (GC12 vs. GC3) and ENC-plot analysis were performed to determine the relative contributions of mutational pressure and natural selection in shaping the codon usage patterns. In neutral plot analysis, a plot regression with a slope of zero indicates no effect of directional mutation pressure (Sueoka, 1988). The ENC-plot (ENC vs. GC3s) was mapped with the scatterplot. If the point lies exactly on the standard curve (corresponding to the ENC values), the determinant of codon preference is mutation pressure (Wright et al., 1990). Simple sequence repeats (SSRs) were searched via MISA v1.01 (Beier et al., 2017) with the following criteria: 10, 5, 4, 3, 3, and 3 repeat units for mono-, di-, tri-, tetra-, penta-, and hexa-nucleotides, respectively. Chloroplast genome similarity was assessed using BLAST Atlas on the GView server (https://server.gview.ca/) with 10 kbp connection windows (Petkau et al., 2010) with *Thuniopsis cleistogama* _L04 genome as a reference. Circos plot of genomic landscape for small genomic variants based on SNP and InDel data was created using the TBtools (Chen et al., 2020). The junction regions between the IR, SSC, and LSC of these plastomes were compared using IRscope online program (Amiryousefi et al., 2018). The divergent regions were visualized using Shuffle-LAGAN mode (Brudno et al., 2003) included in mVISTA v.2.0 (Frazer et al., 2004). To identify polymorphic regions with substantial variability, the aligned sequences were imported in DnaSP v6.12.03 (DNA Sequences Polymorphism) using the sliding window method with a step size of 200 bp and a window length of 600 bp (Rozas et al., 2017).

### Phylogenetic Analyses

We reconstructed the phylogenetic relationships among 18 species representing eight genera in subtribe Coelogyninae based on 24 complete chloroplast (cp) genomes, including five newly sequenced plastomes for each species of *Thuniopsis*, *Thunia* and 19 previously reported plastomes of 16 species downloaded from GenBank database. *Arundina graminifolia* (GenBank accession No. MN171408) was included as outgroup based on previous study (Li et al., 2015). All the genome sequences were aligned using MAFFT v7.313 (Katoh and Standley, 2013) and adjusted manually by BioEdit (Hall, 1999). We used maximum likelihood (ML) and Bayesian inference (BI) methods for phylogenetic analyses. The ML tree was conducted using IQ-TREE v.1.6.12 (Nguyen et al., 2015) and web server (http://iqtree.cibiv.univie.ac.at). The best-fitting nucleotide substitution model TVM + F + R2 was determined using the Akaike Information Criterion (AIC) by ModelFinder (Kalyaanamoorthy et al., 2017) in the IQ-TREE package and 1,000 bootstrap replicates. The Bayesian inference was performed with MrBayes v.3.2.7 (Ronquist et al., 2012), employing the TVM + F + R2 model of nucleotide substitution, as determined by ModelTest-NG 0.1.6 (Darriba et al., 2019). Two independent Markov Chain Monte Carlo (MCMC) runs were performed for 1,000,000 steps with a random starting tree and sampled every 1,000 generations. Each chain started with a random tree, and the first 25% sampled trees were discarded as burn-in to construct a majority-rule consensus tree and to estimate posterior probabilities (PP).

## Results

### Plastome Features of Two Newly Sequenced Orchids

**FIGURE 1:**
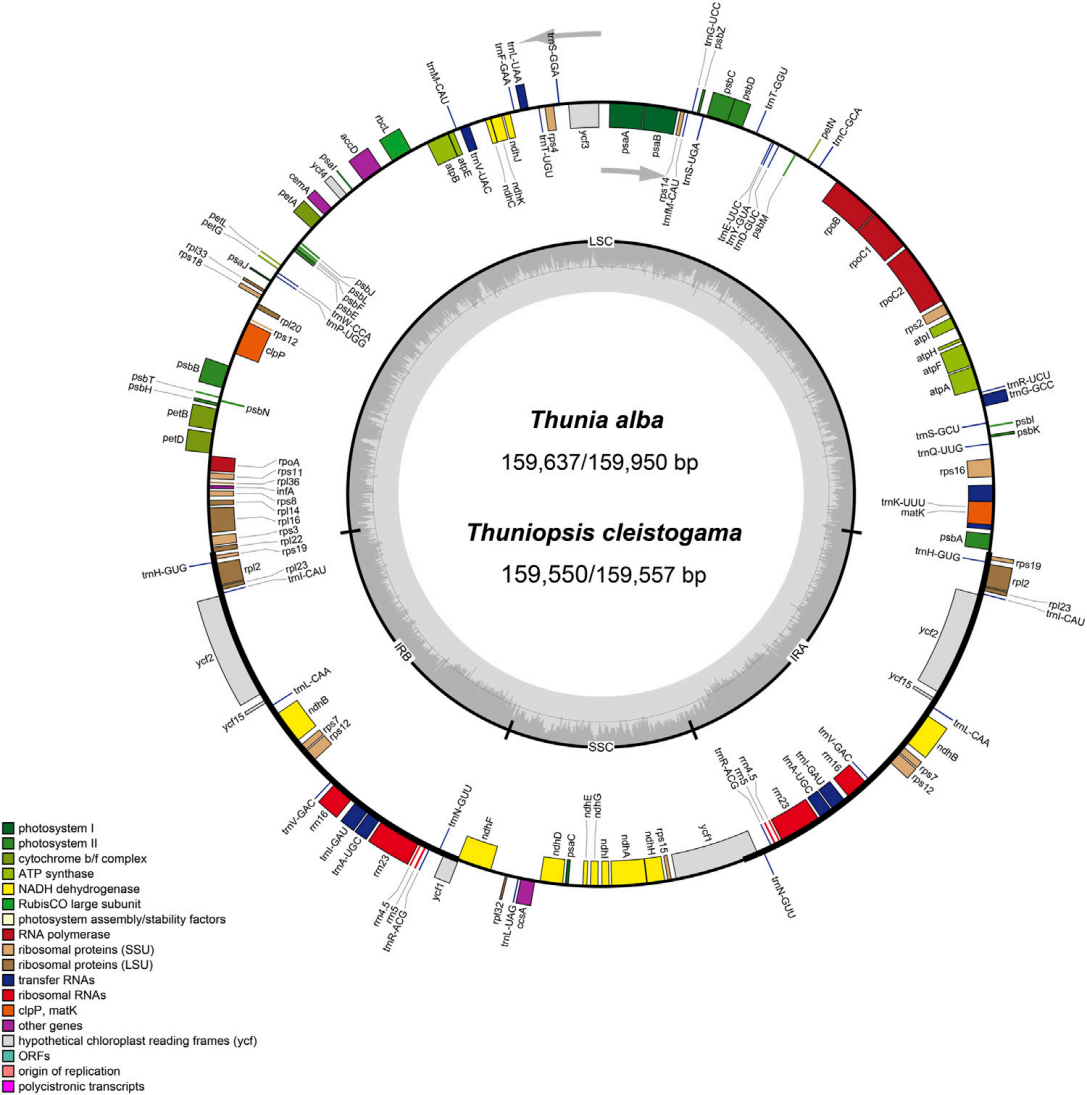
Circular gene map of chloroplast genomes from *Thuniopsis cleistogama* and *Thunia alba*. Genes inside and outside of the circle are transcribed in clockwise and counterclockwise directions, represented with arrows. Bars of different colors indicate different functional groups. LSC, large single copy region; IR, inverted repeats (IRa and IRb); SSC, small single copy region.

We obtained the whole chloroplast genomes of three samples for *Thuniopsis cleistogama* (GenBank accession Nos. OL809657, OL809660 and OL809661) and two samples for *Thunia alba* (GenBank accession Nos. OL809658, and OL809659). As most angiosperms plastid genomes, the newly assembled plastomes exhibited the classical quadripartite circular structure, with two inverted repeat (IRA and IRB) regions separated by a Large Single Copy (LSC) and a Small Single Copy (SSC) by OGDRAW (Figure 1). The average assemblies of the five cp genomes varied in size from 159,550 to 159,950 bp (Table 1). The LSC regions ranged in size from 87,223 to 87,533 bp; the SSC regions varied between 18,858 and 18,870 bp; and the IR regions varied from 26,697 bp to 26,778 bp. These cp genomes of the two taxa show highly syntenic nature in gene content and order. Each of the genomes encoded a total of 135 unique genes, including 89 protein-coding genes (65.93%), 38 tRNA genes (28.15%) and eight rRNA genes (5.29%). A total of 19 genes contained two exons, including 8 tRNA genes (two *trnA*, *trnG*, two *trnI*, *trnK*, *trnL*, *trnV*) and 11 protein coding genes (*atpF*, *ndhA*, two *ndhB*, *petB*, *petD*, two *rpl2*, *rpl16*, *rpoC1*, *rps16*), while the other 4 protein coding genes (*clpP*, *ycf3*, *two rps12*) each contained three exons. Eight protein-coding genes (*ycf2*, *ycf15*, *rps19*, *rpl2*, *rpl23*, *rps7*, *rps12*, and *ndhB*), eight *tRNAs* (*trnH-GUG*, *trnI-CAU*, *trnI-GAU, trnL-CAA*, *trnN-GUU*, *trnR-ACG*, *trnV-GAC*, and *trnA-UGC*), and all four rRNAs (*rrn4.5*, *rrn5*, *rrn16* and *rrn23*) included two copies because of their location at the IR regions. In addition, the genes *ycf1*, *ndhF* were located at the junctions of SSC/IR and *rpl22* was located at the junction of LSC/IR. The *rps12* gene was found to be trans-spliced, consisting of three exons, with a single copy *rps12. e1* located in the LSC region, whereas two copies of *rps12. e2* and *rps12. e3* located in the IRs. The Guanine-Cytosine (GC) content of the IR regions (43.18–43.24%) is higher than that of the LSC (35.03–35.09%) and SSC regions (30.19–30.28%).

**TABLE 1:** Characteristics of chloroplast genomes of *Thuniopsis cleistogama* and its related taxa.

Species/Individuals	*Bletilla formosana*	*Bletilla striata*	*Pleione bulbocodioides*	*Pleione maculata*	*Thunia alba*_L01	*Thunia alba*_L02	*Thuniopsis cleistogama*_L03	*Thuniopsis cleistogama*_L04	*Thuniopsis cleistogama*_L05
GenBank No.	MN562087	MT193723	NC_036342	MW699846	OL809658	OL809659	OL809660	OL809661	OL809657
Size (bp)	159,112	159,491	159,269	158,394	159,637	159,950	159,557	159,557	159,550
LSC (bp)	86,838	87,139	87,121	86,603	87,223	87,533	87,293	87,293	87,288
SSC (bp)	18,672	18,778	18,712	18,499	18,858	18,861	18,870	18,870	18,868
IRs (bp)	26,801	26,787	26,718	26,646	26,778	26,778	26,697	26,697	26,697
Coding (bp)	81,033	81,069	80,943	80,685	80,910	80,913	80,805	80,805	80,805
Noncoding	78,079	78,422	78,326	77,709	78,727	79,037	78,752	78,752	78,745
Number of genes	135	135	135	135	135	135	135	135	135
Protein-coding genes	89	89	89	89	89	89	89	89	89
tRNA genes	38	38	38	38	38	38	38	38	38
rRNA genes	8	8	8	8	8	8	8	8	8
Total GC (%)	37.28	37.17	37.22	37.32	37.26	37.22	37.22	37.22	37.22
LSC (%)	35.12	34.96	35.03	35.18	35.09	35.03	35.09	35.09	35.09
SSC (%)	30.38	30.22	30.30	30.41	30.28	30.27	30.19	30.19	30.19
IR (%)	43.19	43.20	43.21	43.21	43.24	43.24	43.18	43.18	43.18

### Codon Usage Bias Analysis

Codon usage bias (CUB) is an essential feature of genome which provides important information for understanding species evolution. As described by Sharp and Li (1987), relative synonymous codon usage (RSCU) refers to the ratio of its actual usage frequency of a particular codon to expected frequency in the absence of codon usage bias. The RSCU analysis identified totally 80,805–80,913 bp protein-coding genes based on the five cp genomes of *Thuniopsis cleistogama* and *Thunia alba*, accounting for 50.58–50.64% of the entire genome sequence. These genes are encoded in 26,935–26,971 codons. The RSCU value of each codon for their amino acid was calculated (Supplementary Table S1). Among the 21 amino acids (Supplementary Figure S1), 19 amino acids are encoded by two, three, four or six codons with the exception of methionine (Met) and tryptophan (Trp). There are three amino acids: Arginine (Arg), leucine (Leu), and serine (Ser), each are encoded by six different codons. On average, leucine (Leu, encoded by UUA, UUG, CUU, CUC, CUA and CUG) was the most frequent amino acid, comprising 2,808–2,809 (10.4%) of the total number of codons whereas cysteine (Cys, encoded by UGU and UGC) was the least frequently encoded amino acid, with 322–324 codons (1.2%). The results indicated that the majority of preferred codons (RSCU >1) ended in A or U, with the exception of UUG (RSCU = 1.21).

To investigate the extent of codon usage bias in the cp genomes of *Thunia alba* (L01 and L02) and *Thuniopsis cleistogama* (L03, L04 and L05), the effective number of codons (ENC) values were calculated. The detailed information of ENC values was shown in Supplementary Table S2. The ENC values varied from 30.698 to 60.224, with the highest in *Thunia alba*; in contrast, lowest in *Thuniopsis cleistogama*, displaying different trends in codon preferences between the species. Furthermore, we identified only three genes with an ENC value smaller than 35, indicating a low codon usage bias. More specifically, the ENC values of individual genes differ significantly between the two species. For gene *petL*, the ENC value in *Thunia alba* was 42.429, whereas the ENC value in *Thuniopsis cleistogama* was 61. To further explore their differences, the distribution of the ENC values of the coding genes in the genomes was shown in Supplementary Figure S2.

**FIGURE 2:**
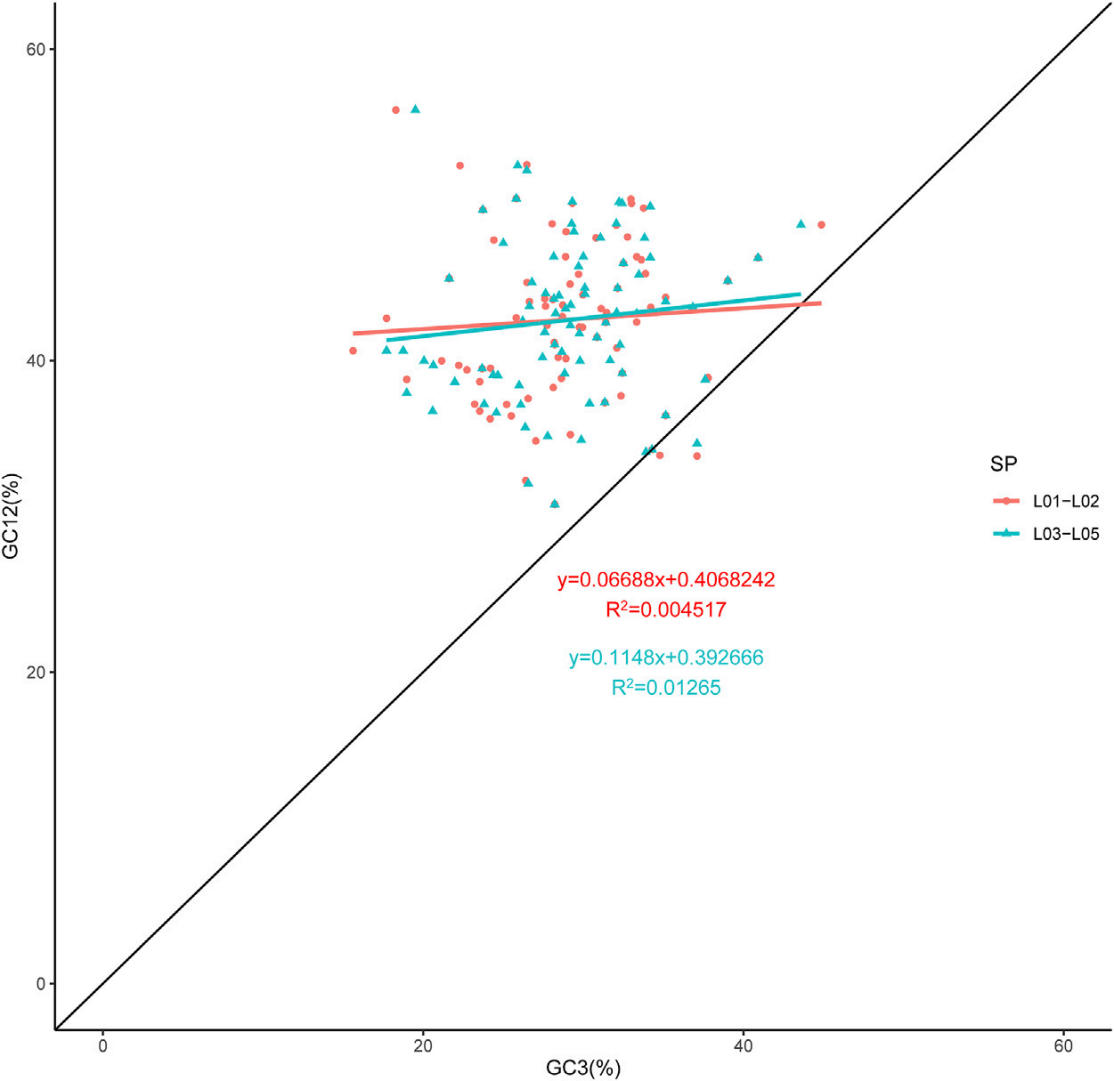
Neutrality analysis performed by plotting GC12 values against GC3 values for the cp genomes of *Thunia alba* (L01 and L02) and *Thuniopsis cleistogama* (L03, L04 and L05). The diagonal line on the neutrality plot shows that the value of GC12 is equal to GC3.

The overall GC content of the genomes was 37.22–37.26%, indicating nearly identical levels among the five chloroplast genomes. As expected, we found that the GC1, GC2 and GC3 contents varied significantly across species and also among genes in the genomes. The greatest difference of GC content was found in GC3 (Supplementary Table S3), which was widely used to better elucidate the codon usage variation. The neutrality plot revealed a weak correlation between GC3 and GC12. As shown in Figure 2, the correlation coefficient was very low, indicating that the GC composition for the three positions of the codon differed. In the neutrality plot of all the genes generated, the slope of the regression line was close to zero, and most plotted points did not lie on or along the diagonal line. These data gave evidence that the codon preference was dominated by natural selection (Sueoka, 1988). Compared to *Thuniopsis cleistogama*, the slope of the regression line for *Thunia alba* was smaller, with the data points almost form a horizontal line. This observation suggested that *Thunia alba* codon usage was more affected by natural selection.

The ENC-plot analysis (Supplementary Figure S3) revealed that only a few points were located on or close to the expected curve, most ENC values lower than expected values were observed to lie below the curve. These results thus confirmed that codon usage preference of the five cp genomes was mainly influenced by natural selection and other factors, while mutation pressure played only a minor role (Wright et al., 1990).

### SSR Analysis

**FIGURE 3:**
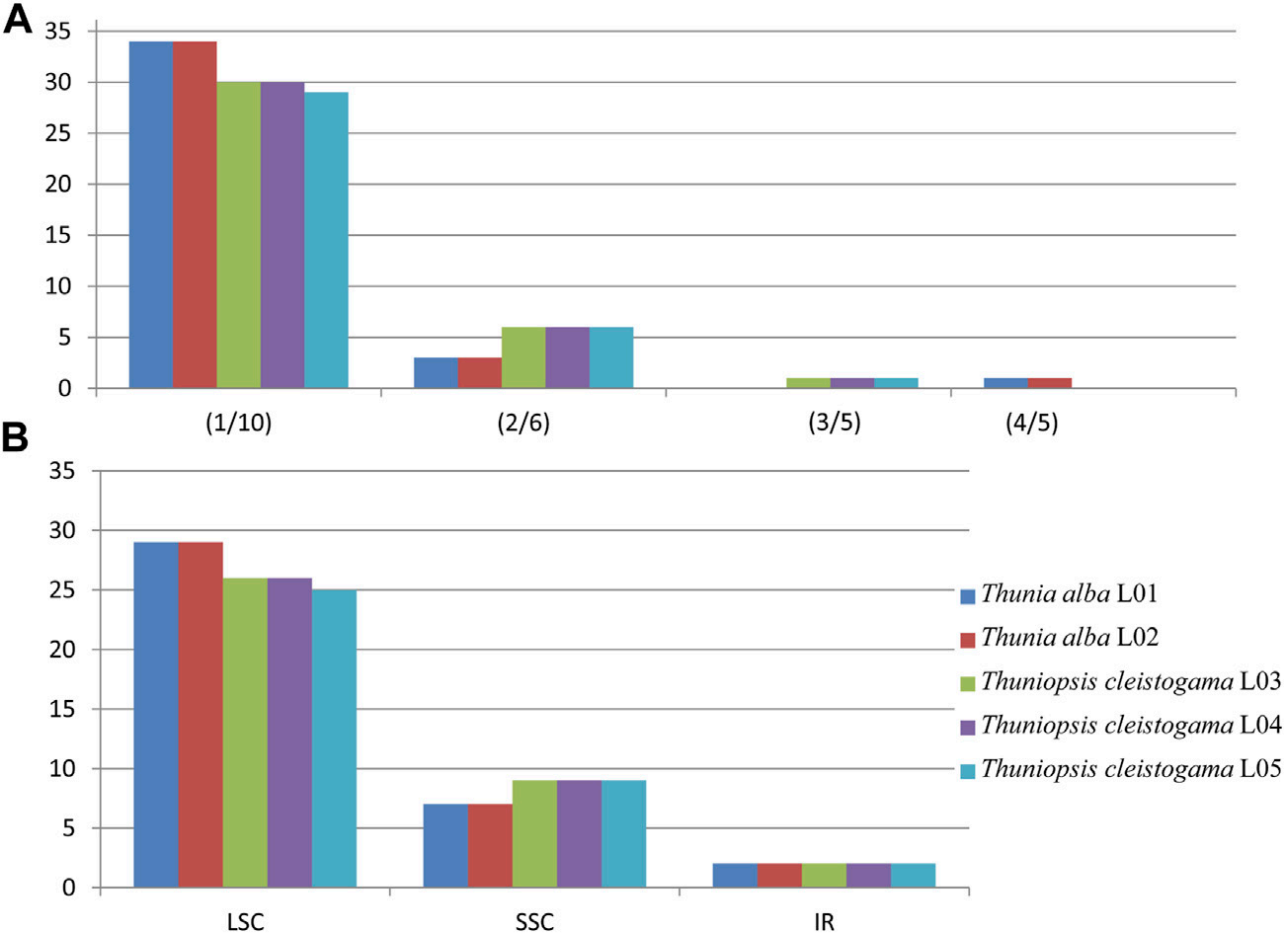
The type and distribution of chloroplast simple sequence repeats (cpSSRs) in the cp genomes from the five individuals of *Thuniopsis cleistogama* and *Thunia alba*. **(A)** Frequencies of cpSSRs identified in different repeat types. **(B)** Number of cpSSRs in different regions.

Simple sequence repeats (SSRs), also known as microsatellites, consist of short tandemly repeated DNA sequences of 1–6 base pair units. Their rapid evolution makes them useful for tracing the evolutionary history of populations and investigating patterns of selection (Pauwels et al., 2012; Asaf et al., 2016), In this investigation, we detected a total of 186 simple sequence repeats (SSRs) with four types (i.e., mono-, di-, tri-, and tetra-nucleotide repeats) for the chloroplast genomes of each species sampled. The details of all cpSSRs identified in these plastomes are represented in Supplementary Table S4. The MISA analysis (Beier et al., 2017) identified 36 to 37 SSRs in each *Thuniopsis* cp genome, whereas 38 SSRs in each *Thunia* cp genome. The tetra-nucleotide SSR was only identified in *Thunia* whereas tri-nucleotide SSR only exists in *Thuniopsis* (Figure 3). In each case, The SSRs varied with the number of repeats for each type depending on the species and individuals examined. The results indicated that the selected SSRs can detect a relatively wide genetic diversity in genomes of different individuals or populations.

Among all SSRs identified across the genomes, mononucleotide repeats were the most common SSRs (29–34, the average percentage of 84.41%), followed by di-nucleotide SSRs, accounted for 12.9% of the total repeats, while tri-nucleotide and tetra-nucleotide SSRs occurred less frequently (1.61, 1.08% of all SSRs, respectively). The majority of SSRs were located in the LSC regions (25–29, the average percentage of 72.58%), followed by SSC regions (7–9, the average percentage of 22.04%). Only two SSRs were identified in IR regions, made up 5.38% of all SSRs. Only one tri-nucleotide SSR (for species *Thuniopsis cleistogama*) and one tetra-nucleotide SSR (for species *Thunia alba*) were identified in IR, made up 5.38% of all SSRs. In addition, SSRs were composed primarily of A or T with an obvious A/T bias in these plastomes. The highest mononucleotide SSRs (89.47%) composed of A/T units. Meanwhile, AT/TA repeats were the most common among di-nucleotide SSRs (A+ T: 7.89%).

### Plastome Visualization With Gview

**FIGURE 4:**
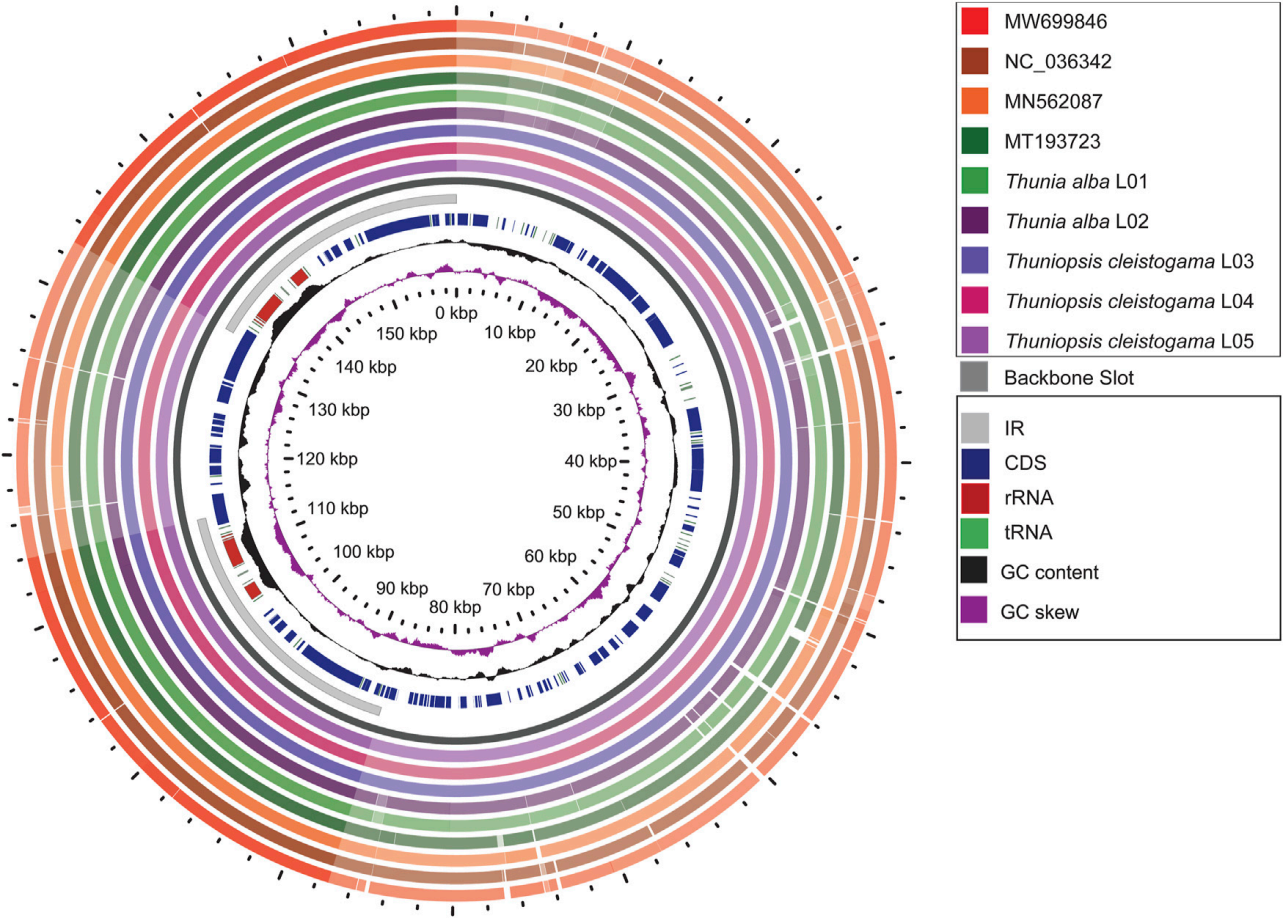
Graphical map shows nine circular plastome assemblies using *Thuniopsis cleistogama*_L04 plastome alignment as a reference. The innermost ring shows the genome size in kbp, followed by GC skew (purple) and GC content (black). The remaining rings denote BLAST comparisons of plastome sequences. From the inner to outer: *Thuniopsis cleistogama*_L05, *Thuniopsis cleistogama*_L04, *Thuniopsis cleistogama*_L03, *Thunia alba*_L02, *Thunia alba*_L01, MT193723; MN562087; NC_036342 and MW699846. The similar and divergent locations are represented by continuous and interrupted track lines, respectively. The lightly screened area stretching from the inner toward the outer circle marks divergent regions with large sequence differences.

To investigate intra-generic and interspecific divergence, a graphical genome map was generated using the Gview tool (Petkau et al., 2010). The overall genome features and structural variations were assessed for the plastomes sequenced and assembled here and previously reported plastomes with *Thuniopsis cleistogama* _L04 genome as a reference. All the nine cp plastomes from six species showed high similarity in genomic structure. However, much higher genetic inconsistency occurred in the LSC and SSC regions compared to the IR regions. No significant difference was detected between individuals or populations of the same species. The variability among species within the same genera was generally not significant than the diversification across species in different genera (Figure 4).

### Exploration of SNPs and InDels

**FIGURE 5:**
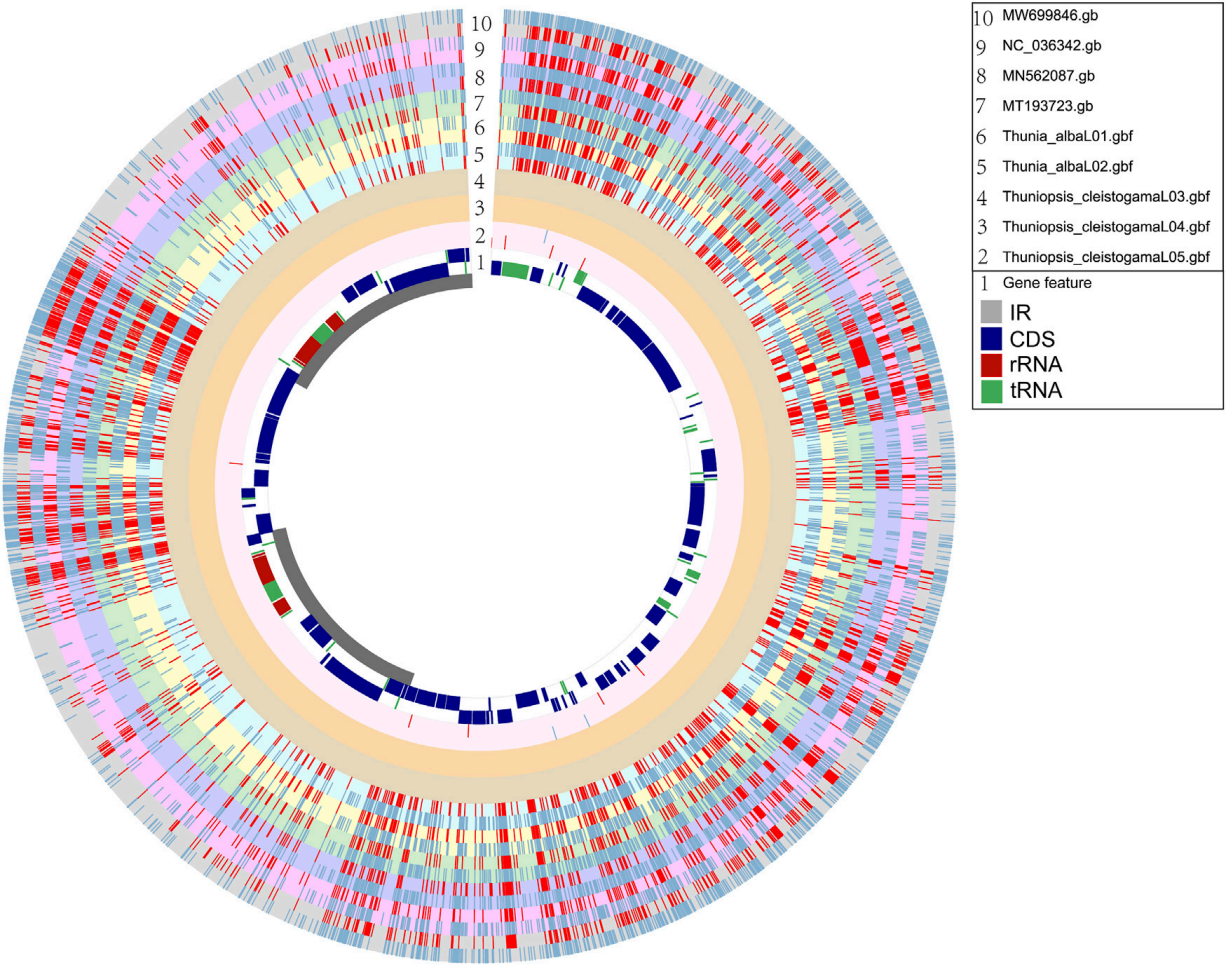
Circos plot based on complete plastome alignment of newly sequenced plastomes (*Thuniopsis cleistogama* and *Thunia alba*) and relatives available at NCBI (MN562087, MT193723, MW699846 and NC_036342). Ring “1” shows the reference plastome alignment of *Thuniopsis cleistogama_*L04, with coding genes labeled in dark blue, rRNAs in red and tRNAs in green. Inner ring, gray: IR region, white: LSC and SSC regions. The rings “2–10” indicate SNPs and InDels, respectively. Blue shadows denote the locations of SNPs; Red shadows denote the locations of InDels. The height of columns in rings “2–10” represents the relative number of polymorphic loci. From outside to inside, ring “10” shows MW699846 (gray); ring “9” shows NC_036342 (pink); ring “8” shows MN562087 (blue); ring “7” shows MT193723 (light green); ring “6” shows *Thunia alba_*01 (light yellow); ring “5” shows *Thunia alba_*02 (light blue). ring “4” shows *Thuniopsis cleistogama*_03 (dusty gray); ring “3” shows *Thuniopsis cleistogama* 04 (ochre); ring “2” shows *Thuniopsis cleistogama*_05 (light pink). Two contiguous rings with same color indicate positive and negative strand, respectively.

For estimating genetic variations across the genomes of five newly sequenced samples and four plastomes available in GenBank, we identified single nucleotide polymorphisms (SNPs) and insertions/deletions (InDels) in relation to the reference genome. The Circos plot represents the distribution pattern of SNPs and InDels in the genic regions of the nine plastomes (Figure 5). The SNPs and InDels that detected for each plastome alignment were statistically analyzed (Supplementary Table S5). The polymorphisms identified from our alignment were not evenly distributed across the segments and varied clearly across different taxonomic levels (intergeneric and intrageneric). Compared with the reference genome of *Thuniopsis cleisostogama*_L04, genetic diversity among individuals of the same species are relatively low. We found only 3 SNPs and 8 InDels in the sample of *T. cleisostogama* L05. In comparison, the number of SNPs detected in two individuals of *Thunia alba* ranged from 2,006 to 2,044, InDels ranged from 1,358 to 1,446. The number of SNPs detected in *Bletilla* taxa ranged from 1,958 to 2,026; InDels ranged from 1,195 to 1,291. The number of SNPs detected in *Pleione* taxa ranged from 1,917 to 1,972; InDels ranged from 1,363 to 1,518. Totally 11,923 SNPs and 8,171 InDels were identified among these plastomes (rings “2–10”). The average numbers of SNPs and InDels are 1,987 and 1,362, respectively, with the ratio 1.459: 1, indicating that SNPs represent the most common type of DNA polymorphism in these genomes. In general, SNPs and InDels were spread over the genomes with a similar distribution pattern. The majority of these polymorphisms were found in the noncoding and intergenic regions of the genomes. Furthermore, genes at the IR and SSC junctions showed comparably higher variations. Among more than 7,000 variant sites, only more than 1,400 were located in the coding regions. Therefore, the overall variants of non-coding regions are higher than those of coding regions.

### Comparison of Sequences Flanking IR/SC Junctions

**FIGURE 6:**
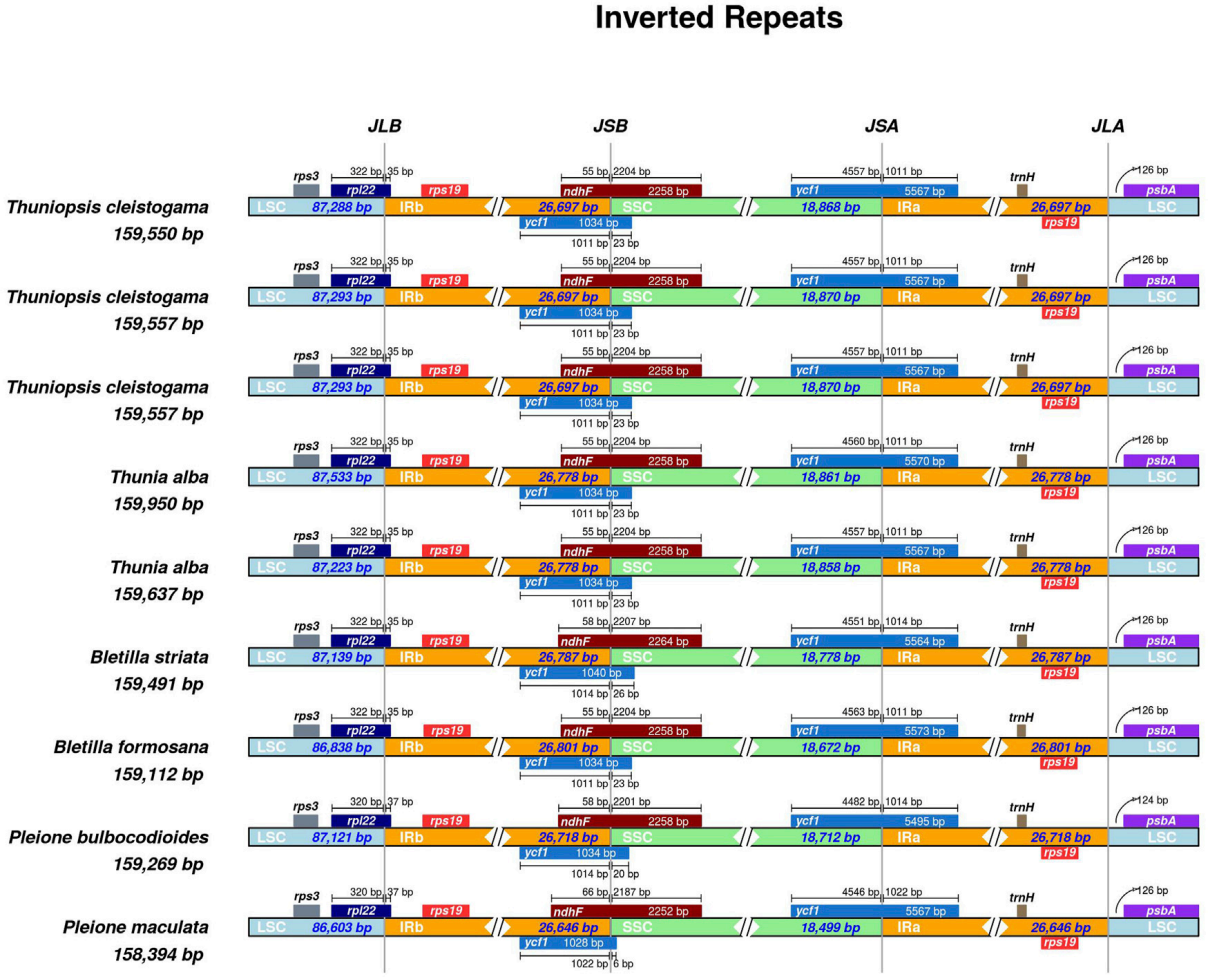
Comparison of the borders of large single-copy (LSC), small single-copy (SSC), and inverted repeat (IR) regions among the nine cp genomes. The numbers above the gene features denote the distance between the gene borders, either the start or end of genes and the junction sites.

The LSC/IR and SSC/IR borders of the three cp genomes of *Thuniopsis cleistogama* were compared with the two cp genomes of *Thunia alba*, and of the published cp genomes of four other closely related species (*Bletilla formosana*, *B. striata*, *Pleione bulbocodioides* and *P. maculata*). As shown in Figure 6, the *rps*3 gene was situated in the LSC region. The *ycf1* and *ndhF* genes were located at the junction of the SSC/IR borders, while *rpl22* was mainly located in the boundary of the LSC/IRb junction. Two copies of the *rps*19 genes were present in the IRa and IRb regions, near the LSC/IR borders. In all the cp genomes at the SSC/IR borders, the IR extended into the *ycf1* gene to create a long ycf1 gene of 1,028–1,034 bp. Two copies of the ycf1 genes crossed the SSC/IRa and SSC/IRb borders, respectively. As a whole, the junctions between the IR and LSC/SSC regions slightly varied among these cp genomes. The *rpl22* gene extended from the LSC regions into the IRb regions by 35 bp in *Thuniopsis cleistogama*, *Thunia alba*, *Bletilla formosana* and *B. striata*. This distance was 37 bp away from the LSC/IRb border in *Pleione bulbocodioides* and *P. maculata*. The functional *ycf1* gene spanned the IRa and SSC regions, with 4,557 to 4,560 bp sequences situated at SSC region in *Thuniopsis cleistogama* and *Thunia alba*, and 4,551 to 4,563 bp in *Bletilla formosana* and *B. striata*, 4,482 to 4,546 bp in *Pleione bulbocodioides* and *P. maculata*. The sizes of the fragments located in the IRa regions were 1,011 bp in *Thuniopsis cleistogama* and *Thunia alba*, 1,011 to 1,014 bp in *Bletilla formosana* and *B. striata*, 1,014 to 1,022 bp in *Pleione bulbocodioides* and *P. maculata*, respectively. The *ndhF* gene was situated at the IRb/SSC boundary, with 55 bp sequences situated at the IRb region in *Thuniopsis cleistogama*, *Thunia alba* and *Bletilla formosana*, the comparable region in *Bletilla striata* and *Pleione bulbocodioides* is 58 bp long, whereas in *P. maculata*, with 66 bp apart from the SSC/IRb border.

### Sequence Divergence Analysis and Identification of Polymorphic Regions

**FIGURE 7:**
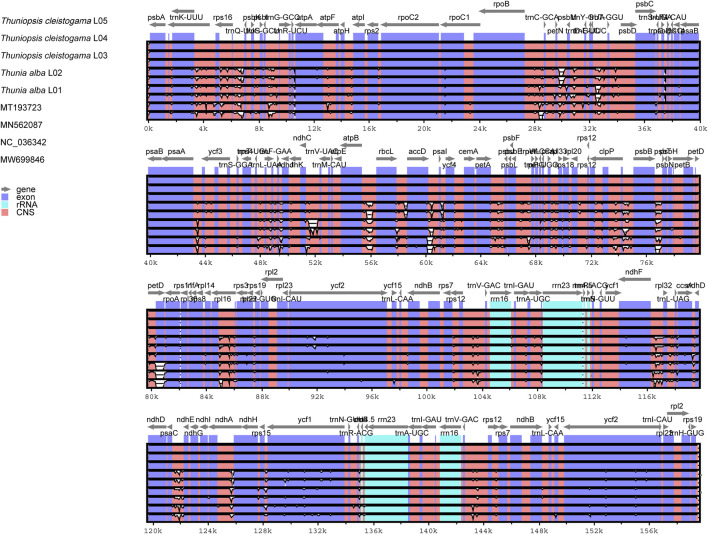
Comparison of nine cp genomes of six taxa using mVISTA program with *Thuniopsis cleistogama*_L04 cp genome as a reference. The top gray arrows above the alignment indicate genes and their orientation. A cut-off of 70% was used for the plots. Genome regions are color coded. Blue and red areas indicate protein-coding regions and the conserved non-coding sequences (CNS) regions, respectively.

To elucidate the level of the plastome divergence, the complete chloroplast genomes of *Thuniopsis* were compared and plotted using mVISTA (Frazer et al., 2004) by aligning the nine cp genomes with the reference *T. cleistogama* L04 genome (Figure 7). The noncoding regions showed a significantly higher degree of sequence divergence than that observed in the coding regions. The LSC and SSC regions were more divergent between species than the two IR regions. Most sequence variations were found concentrated in the intergenic regions, which exhibited noticeably higher divergence than the other regions.

**FIGURE 8:**
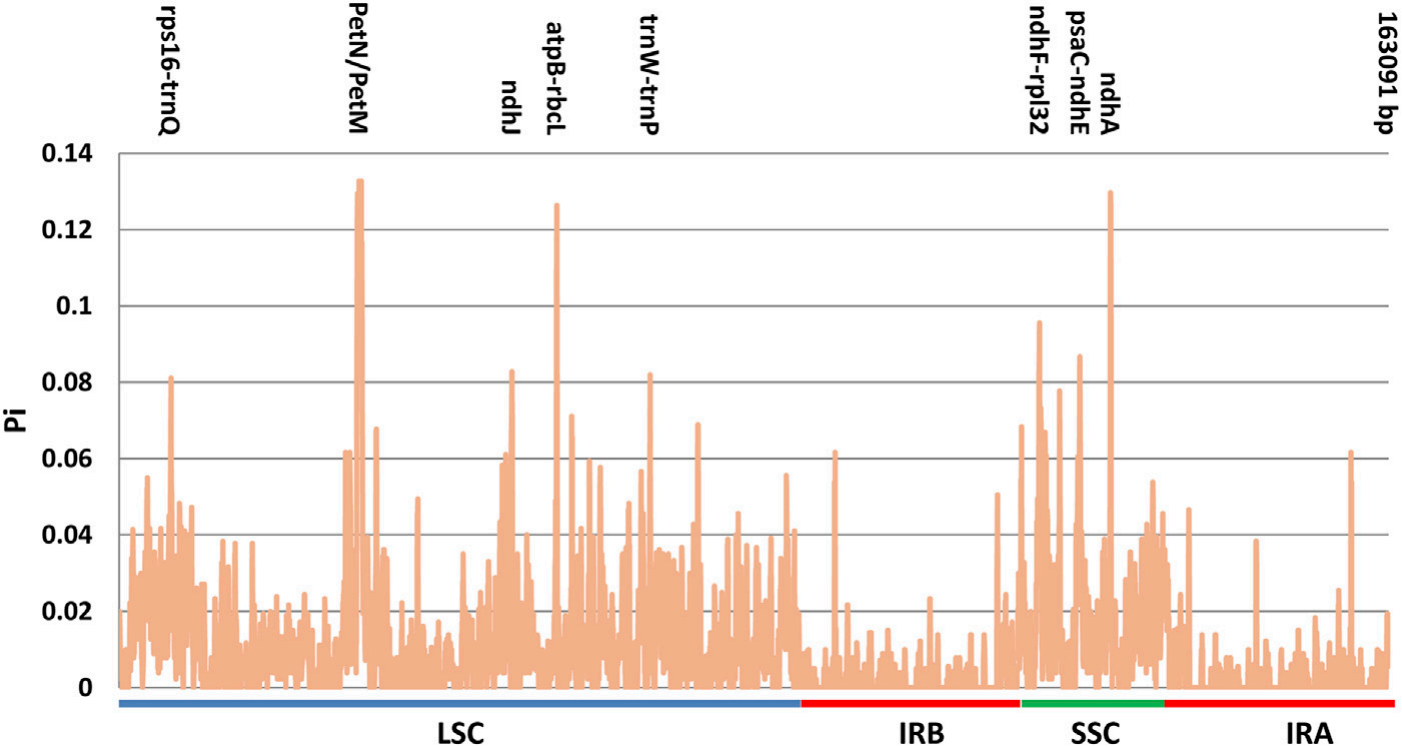
Nucleotide diversity (Pi) values among the nine cp genomes.

In order to detect highly variable regions, polymorphic sites and nucleotide variability (polymorphism information, Pi) were calculated using a sliding window analysis (Figure 8). Among the nine plastomes, Pi values ranged from 0 to 0.13278 (*petN-petM*). The sequences were more conserved in the IR regions than in the LSC and SSC regions in all plastomes. The nucleotide variability was higher in the SSC (Pi = 0.0190) and LSC (Pi = 0.0135) regions, when compared to IR regions (Pi = 0.0031), which had a much lower nucleotide diversity. The Pi values in non-coding regions (with an average value of 0.019448) showed comparably higher divergence levels than the coding regions (with an average value of 0.01008).

In the LSC region, one intergenic region (*petN-petM*) showed the highest Pi value of 0.13278, followed by the *atpB-rbcL* with Pi value of 0.12639. In the SSC region, *ndhA* showed a higher evolution rate compared with other genes, with Pi value of 0.12972. Eight hypervariable regions among the cp genomes were recognized as hotspot regions with nucleotide diversity >0.08. These regions were *petN-petM* (0.13278), *ndhA* (0.12972), *atpB-rbcL* (0.12639), *ndhF-rpl32* (0.09556), *psaC-ndhE* (0.08667), *ndhJ* (0.08278), *trnW-trnP* (0.08194) and *rps16-trnQ* (0.08111). Among them, *ndhF-rpl32* spanned the SSC/IRb boundary. *ndhA* and *psaC-ndhE* were situated in the SSC region, whereas five of eight were located in the LSC region (*petN-petM*, *atpB-rbcL*, *ndhJ*, *trnW-trnP* and *rps16-trnQ*).

Plastome sequences varied most in the intergenic spacer (IGS) regions. Six regions showed Pi values higher than 0.08 and all of these regions were located in the IGS region, e.g., *petN-petM*, *atpB-rbcL*, *ndhF-rpl32*, *psaC-ndhE*, *trnW-trnP* and *rps16-trnQ*. Two genes: *ndhJ* of the LSC region and *ndhA* of the SSC region present higher nucleotide variability than the mean gene. These divergent hotspots show potential value for the development of molecular markers for phylogenetic and phylogeographic analysis.

### Phylogenetic Analyses

**FIGURE 9:**
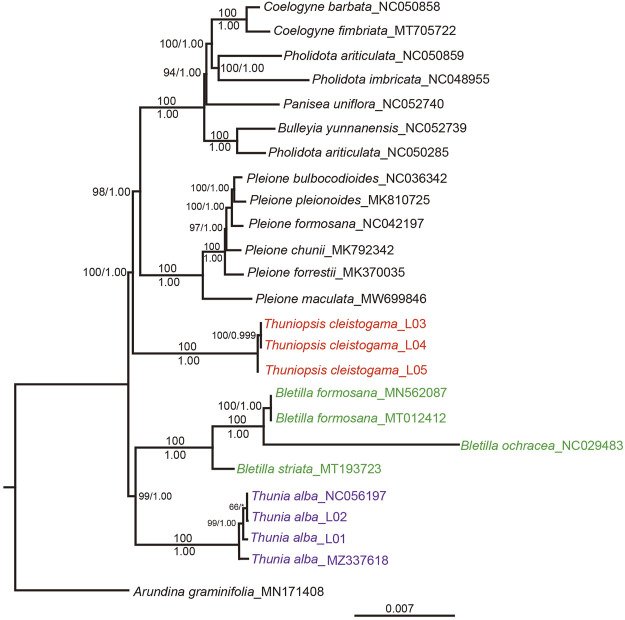
The ML phylogeny of *Thuniopsis* and its closely related genera in subtribe Coelogyninae based on complete plastomes. The obtained bootstrap values (BS) and Bayesian inference posterior probabilities (PP) are marked at the tree node (BS/PP). *Thuniopsis* species are highlighted in red, *Thunia* species are highlighted in purple, and *Bletilla* species are highlighted in green.

The ML and BI analyses of the complete chloroplast genomes generated consistent topologies. The ML topology was selected for discussion, with ML bootstrap (MLBS) and posterior probabilities (PP) values are given near nodes (Figure 9). The sister relationship between genera *Bletilla* and *Thunia* was recovered (PP = 1.00; BS = 99). *Thunia* represented by *T. alba*, and *Bletilla* represented by *B*. *formosana*, *B*. *striata* and *B*. *ochracea*. The same clade was seen in previous analyses (Li et al., 2015). All phylogenetic analyses consistently indicated that three sampled specimens of *Thuniopsis cleistogama* formed a well-supported monophyletic lineage (PP = 1.00; BS = 100), sister to the clade containing species of the rest genera examined within the subtribe Coelogyninae, including *Bulleyia*, *Coelogyne*, *Panisea*, *Pholidota* and *Pleione*. These results indicated that *Thuniopsis* formed an independent lineage that genetically separated from the rest genera in Coelogyninae.

## Discussion

### Comparative Analysis of the Chloroplast Genomes

Generally, our results showed d that the chloroplast genomes from six species, representing the four genera (*Bletilla*, *Thunia*, *Thuniopsis* and *Pleione*) were rather similar in terms of structural organization, gene content and gene order. Genome annotation identified a total of 135 unique genes, consisting of 89 protein-coding genes, 38 tRNA genes, and eight rRNA genes in each genome (Table 1). Nevertheless, the cp genome size varied within each genus, species, and even within individual from the same species, ranging from 158,394 in *P*. *maculata* to 159,269 bp to 159,950 bp in *Thunia alba*_L02. The LSC regions ranged in size from 86,603 bp in *P*. *maculata* to 87,533 bp in *Thunia alba*_L02; the SSC regions ranged from 18,499 bp in *P*. *maculata* to 18,870 bp in *Thuniopsis cleistogama*_L03 and L04, and the IR regions ranged from 26,646 bp in *P*. *maculata* to 26,801 bp in *Bletilla formosana*. In comparison with other species of related genera, *Thuniopsis cleistogama* possesses a relatively larger SSC region (18,868–18,870 bp).

Genomic DNA base composition (GC content) is predicted to significantly affect genome functioning (Šmarda et al., 2014). Our analysis showed that the GC contents varied among different genomes and different regions within a genome. When compared with the LSC (34.96–35.12%) and SSC (30.19–30.41%) regions, we observed significantly higher GC contents in the IR regions (43.18–43.24%). The high GC content could be caused by the presence of eight rRNA genes with high GC content in these regions. The result was consistent with previous studies (Darshetkar et al., 2019; Wu et al., 2020). GC content generally showed a quadratic relationship with genome size. Interestingly, with respect to its smallest genome (158,394 bp), *Pleione maculata* genome (MW699846) has a relatively higher GC content (37.32%) than expected. According to Šmarda et al. (2014), in monocots, increased GC content was associated with increased tolerance and ability to grow in seasonally cold and/or dry climates.

Although the cp genomes of *Thuniopsis* and its related genera showed extremely conserved structure, slight variations were found at the LSC/IR and IR/SSC boundaries of the plastomes. Compared with the LSC/IR border, the IR/SSC border displayed higher variations among species within the four genera (Figure 6). The relatively higher divergence was seen in genes *ycf1* and *ndhF*, which might play an important role in the evolutionary of the cp genome of these species.

Consistent with the diversity patterns found in most angiosperms (Lu et al., 2017; Lee et al., 2019; Zheng et al., 2020), mVISTA analysis (Figure 7) has revealed that sequence variability in non-coding regions was greater than that in coding regions. Overall, sequence divergence was concentrated in the non-coding region, and the sequence divergence in the protein-coding regions (CDSs) was limited. Further, the cp genome sequence comparison has detected large variability in the conserved noncoding sequences regions (CNS). The diversity in CNS is nonrandom, with variants shared across different genera. Many studies have found that CNSs are enriched significantly in regulatory sequence elements. CNSs may have roles in the evolution and some critical biological function (Burgess and Freeling, 2014; Hettiarachchi et al., 2014; Xie et al., 2018).

The nucleotide diversity (Pi) values also indicated the conserved nature of the IR regions (Figure 8). The sequences in the IR regions were more conserved as compared to the LSC and SSC regions. In particular, intergenic regions (IGS) showed significantly higher variability than that in genic regions. Eight highly variable regions were identified, which show high potential value for future DNA barcoding and phylogenetic reconstruction. They are six intergenic markers (*petN-petM*, *atpB-rbcL*, *ndhF-rpl32*, *psaC-ndhE*, *trnW-trnP* and *rps16-trnQ*) and two genic makers (*ndhA*, *ndhJ*).

### Phylogenetic Relationships of *Thuniopsis* and Related Genera

As for the cp genomes newly sequenced in this study, three examined accessions of *Thuniopsis cleistogama* were resolved into a highly supported monophyletic lineage. All four accessions of *Thunia alba* including two accessions obtained from GenBank clustered together as expected. The phylogenetic relationship of *Thuniopsis* and its closely related genera was basically congruent with those of previous studies but found strong support.

In the previous phylogenies by Li et al. (2015) based on partial DNA markers (ITS, *mat*K and *trnL*), *Thuniopsis* formed a poorly supported sister clade to the *Bletilla, Dilochia* and *Thunia* group, with rather low ML bootstrap value of 62%. In this study inferred from the complete plastomes, *Thuniopsis* and *Thunia-Bletilla* were strongly supported as successively diverging lineages. *Bletilla* and *Thunia* were grouped into one clade in both trees with high bootstrap values. The main lineages investigated here are clearly classified with a better resolution. Currently, we were unable to obtain the sequences for genus *Dilochia*. *Dilochia* represented the sister group of *Thunia* in former study (weakly supported). More extensive cp genome sampling is necessary to further illustrate the relationships of species in subtribe Coelogyninae.

In addition to its significant genetic divergence, several studies have suggested that *Thuniopsis* exhibits unique morphological features that differentiate it from other members of Coelogyninae (Li et al., 2015; Kang et al., 2019; Kurzweil et al., 2020). *Thuniopsis* is superficially similar to *Thunia* in the elongate leafy stem, carrying distichous, soft and plicate leaves, but can be easily distinguished from the latter by having prominent subterranean corms; much smaller and spurless flowers; prominent stigma and bilobed rostellum. By contrast, *Thunia* is characterized by its distinctly fleshy, cane-like stems; large and showy flowers. *Thuniopsis* grows in comparatively dry and hot environment. Normally, its stem and leaves of the plant die annually and become dormant during the winter months. The taxonomic status of the genus *Thuniopsis* was confirmed by both molecular analyses and morphological characters*.* The complete chloroplast genomes were proved to be more informative than cp DNA fragments in revealing the phylogeny of Coelogyninae.

## Conclusion

In the present study, the complete cp genomes for *Thuniopsis cleistogama* were determined for the first time and compared with its closely related species in subtribe Coelogyninae. The ENC-plot and neutrality analysis indicated natural selection was the major driving force shaping the codon usage pattern. Comparative analysis of these cp genome sequences revealed conserved genome structure, gene content, and gene order. However, significant divergent sequence hot spots were detected by multiple comparisons. Simple sequence repeats (SSRs), Single nucleotide polymorphism (SNP) as well as insertion/deletion (InDel) provided abundant polymorphisms to evaluate the level of nucleotide divergence of plastomes among genera and species. Sequences in two IR regions were more conserved than those in the LSC and SSC regions. Unsurprisingly, we found that sequence variation in non-coding regions was more divergent than in coding regions. Specifically, most of the polymorphic sites occur in the intergenic regions. Eight regions with high-level polymorphism were uncovered with the potential use as molecular markers. The Phylogenetic analyses achieved well-supported resolution of relationships among all major clades. *Thuniopsis* was resolved as a monophyletic lineage, separate from other groups of Coelogyninae. Our results justify the taxonomic proposal that recognizes *Thuniopsis* as an independent genus. The findings and genomic resources presented in this study may contribute to future research on systematic analysis, genetic diversity and evolutionary patterns of the family Orchidaceae.

## Data Availability

The datasets presented in this study can be found in online repositories. The names of the repository/repositories and accession number(s) can be found in the article/Supplementary Material.

## Supplementary Material

Supplementary Figure S1 Amino acid frequencies within the cp genomes from three *Thuniopsis cleistogama* individuals and two *Thunia alba* individuals. Colors correspond to codons listed underneath the columns. Rows in each column diagram represent the number of groups.

Supplementary Figure S2 Radar chart showing the ENC values of the coding genes in the cp genomes of *Thunia alba* (L01 and L02) and *Thuniopsis cleistogama* (L03, L04 and L05).

Supplementary Figure S3 ENC plot showing the relationship between ENC values and GC3s for the cp genomes of Thunia *alba* (L01 and L02) and *Thuniopsis cleistogama* (L03, L04 and L05). The continuous curve represents the expected codon usage if GC compositional constraints alone account for codon usage bias.

Supplementary Table S1 Codon frequencies and relative synonymous codon usage (RSCU) values of the cp genomes of *Thuniopsis* and *Thunia*.

Supplementary Table S2 The ENC values of the coding genes in the cp genomes of *Thunia alba* (L01 and L02) and *Thuniopsis cleistogama* (L03, L04 and L05).

Supplementary Table S3 The GC contents of three positions of codons (GC1, GC2 and GC3) in the cp genomes of *Thunia alba* (L01 and L02) and *Thuniopsis cleistogama* (L03, L04 and L05.

Supplementary Table S4 Simple sequence repeats (SSRs) detected in the cp genomes from three *Thuniopsis cleistogama* individuals and two *Thunia alba* individuals.

Supplementary Table S5 The statistics of SNPs and InDels identified for each plastome alignments between *Thuniopsis* and *Thunia* species presented here and four plastomes available from NCBI database MT193723; MN562087; NC036342 and MW699846) with *T. cleistogama*_L04 as a reference.

## References

[B1] Amiryousefi A. Hyvönen J. Poczai P. (2018). IRscope: an Online Program to Visualize the junction Sites of Chloroplast Genomes. Bioinformatics 34, 3030–3031. 10.1093/bioinformatics/bty220 29659705

[B2] Asaf S. Khan A. L. Khan A. R. Waqas M. Kang S.-M. Khan M. A. (2016). Complete Chloroplast Genome of *Nicotiana Otophora* and its Comparison with Related Species. Front. Plant Sci. 7, 843. 10.3389/fpls.2016.00843 27379132 PMC4906380

[B3] Beier S. Thiel T. Münch T. Scholz U. Mascher M. (2017). MISA-web: a Web Server for Microsatellite Prediction. Bioinformatics 33, 2583–2585. 10.1093/bioinformatics/btx198 28398459 PMC5870701

[B4] Brudno M. Do C. B. Cooper G. M. Kim M. F. Davydov E. Program N. C. S. (2003). LAGAN and Multi-LAGAN: Efficient Tools for Large-Scale Multiple Alignment of Genomic DNA. Genome Res. 13, 721–731. 10.1101/gr.926603 12654723 PMC430158

[B5] Burgess D. Freeling M. (2014). The Most Deeply Conserved Noncoding Sequences in Plants Serve Similar Functions to Those in Vertebrates Despite Large Differences in Evolutionary Rates. Plant Cell 26, 946–961. 10.1105/tpc.113.121905 24681619 PMC4001403

[B6] Chan P. P. Lowe T. M. (2019). tRNAscan-SE: Searching for tRNA Genes in Genomic Sequences. Methods Mol. Biol. 1962, 1–14. 10.1007/978-1-4939-9173-0_1 31020551 PMC6768409

[B7] Chen C. Chen H. Zhang Y. Thomas H. R. Frank M. H. He Y. (2020). TBtools: an Integrative Toolkit Developed for Interactive Analyses of Big Biological Data. Mol. Plant 13, 1194–1202. 10.1016/j.molp.2020.06.009 32585190

[B8] Chen S. Zhou Y. Chen Y. Gu J. (2018). Fastp: an Ultra-fast All-In-One FASTQ Preprocessor. Bioinformatics 34, i884–i890. 10.1093/bioinformatics/bty560 30423086 PMC6129281

[B9] Darriba D. Posada D. Kozlov A. M. Stamatakis A. Morel B. Flouri T. (2019). ModelTest-NG: A New and Scalable Tool for the Selection of DNA and Protein Evolutionary Models. Mol. Biol. Evol. 37, 291–294. 10.1093/molbev/msz189 PMC6984357 31432070

[B10] Darshetkar A. M. Datar M. N. Tamhankar S. Li P. Choudhary R. K. (2019). Understanding Evolution in Poales: Insights from Eriocaulaceae Plastome. PLoS One 14, e0221423. 10.1371/journal.pone.0221423 31430346 PMC6701780

[B11] Dierckxsens N. Mardulyn P. Smits G. (2017). NOVOPlasty: De Novo Assembly of Organelle Genomes from Whole Genome Data. Nucleic Acids Res. 45, e18. 10.1093/nar/gkw955 28204566 PMC5389512

[B12] Frazer K. A. Pachter L. Poliakov A. Rubin E. M. Dubchak I. (2004). VISTA: Computational Tools for Comparative Genomics. Nucleic Acids Res. 32, W273–W279. 10.1093/nar/gkh458 15215394 PMC441596

[B13] Greiner S. Lehwark P. Bock R. (2019). OrganellarGenomeDRAW (OGDRAW) Version 1.3.1: Expanded Toolkit for the Graphical Visualization of Organellar Genomes. Nucleic Acids Res. 47, W59–W64. 10.1093/nar/gkz238 30949694 PMC6602502

[B14] Hall T. A. (1999). BioEdit: a User-Friendly Biological Sequence Alignment Editor and Analysis Program for Windows 95/98/NT. Nucleic Acids Symp. Ser. 41, 95–98.

[B15] Hettiarachchi N. Kryukov K. Sumiyama K. Saitou N. (2014). Lineage-specific Conserved Noncoding Sequences of Plant Genomes: Their Possible Role in Nucleosome Positioning. Genome Biol. Evol. 6, 2527–2542. 10.1093/gbe/evu188 25364802 PMC4202324

[B16] Kalyaanamoorthy S. Minh B. Q. Wong T. K. F. Von Haeseler A. Jermiin L. S. (2017). ModelFinder: Fast Model Selection for Accurate Phylogenetic Estimates. Nat. Methods 14, 587–589. 10.1038/nmeth.4285 28481363 PMC5453245

[B17] Kang D.-H. Cho S.-H. Ong H. G. Ling S. M. Kyaw N. O. Kim Y.-D. (2019). Two New Generic Records in the Orchid flora of Myanmar. Korean J. Pl. Taxon 49, 96–99. 10.11110/KJPT.2019.49.1.96

[B18] Katoh K. Standley D. M. (2013). MAFFT Multiple Sequence Alignment Software Version 7: Improvements in Performance and Usability. Mol. Biol. Evol. 30, 772–780. 10.1093/molbev/mst010 23329690 PMC3603318

[B19] Kearse M. Moir R. Wilson A. Stones-Havas S. Cheung M. Sturrock S. (2012). Geneious Basic: an Integrated and Extendable Desktop Software Platform for the Organization and Analysis of Sequence Data. Bioinformatics 28, 1647–1649. 10.1093/bioinformatics/bts199 22543367 PMC3371832

[B20] Kurzweil H. Ormerod P. Schuiteman A. (2020). The Long-Lost Myanmar Endemic *Arundina subsessilis* (Orchidaceae) Found Congeneric with the Recently Described Chinese *Thuniopsis cleistogama* . Gbs 72, 97–107. 10.26492/gbs72(1).2020-09

[B21] Langmead B. Salzberg S. L. (2012). Fast Gapped-Read Alignment with Bowtie 2. Nat. Methods 9, 357–359. 10.1038/nmeth.1923 22388286 PMC3322381

[B22] Lee S.-R. Kim K. Lee B.-Y. Lim C. E. (2019). Complete Chloroplast Genomes of All Six *Hosta* Species Occurring in Korea: Molecular Structures, Comparative, and Phylogenetic Analyses. BMC Genomics 20, 833. 10.1186/s12864-019-6215-y 31706273 PMC6842461

[B23] Li L. Ye D.-P. Niu M. Yan H.-F. Wen T.-L. Li S.-J. (2015). *Thuniopsis*: a New Orchid Genus and Phylogeny of the Tribe Arethuseae (Orchidaceae). PLoS One 10, e0132777. 10.1371/journal.pone.0132777 26244769 PMC4526666

[B24] Lu R.-S. Li P. Qiu Y.-X. (2017). The Complete Chloroplast Genomes of Three *Cardiocrinum* (Liliaceae) Species: Comparative Genomic and Phylogenetic Analyses. Front. Plant Sci. 7, 2054. 10.3389/fpls.2016.02054 28119727 PMC5222849

[B25] Nguyen L.-T. Schmidt H. A. von Haeseler A. Minh B. Q. (2015). IQ-TREE: A Fast and Effective Stochastic Algorithm for Estimating Maximum-Likelihood Phylogenies. Mol. Biol. Evol. 32, 268–274. 10.1093/molbev/msu300 25371430 PMC4271533

[B26] Pauwels M. Vekemans X. Godé C. Frérot H. Castric V. Saumitou‐Laprade P. (2012). Nuclear and Chloroplast DNA Phylogeography Reveals Vicariance Among European Populations of the Model Species for the Study of Metal tolerance, Arabidopsis halleri(Brassicaceae). New Phytol. 193, 916–928. 10.1111/j.1469-8137.2011.04003.x 22225532

[B27] Petkau A. Stuart-Edwards M. Stothard P. van Domselaar G. (2010). Interactive Microbial Genome Visualization with GView. Bioinformatics 26, 3125–3126. 10.1093/bioinformatics/btq588 20956244 PMC2995121

[B28] Pridgeon A. M. Cribb P. J. Chase M. W. Rasmussen F. N. (2005). Genera Orchidacearum. New York: Oxford University Press. *Epidendroideae (part one)* .

[B29] Rice P. Longden I. Bleasby A. (2000). EMBOSS: The European Molecular Biology Open Software Suite. Trends Genet. 16, 276–277. 10.1016/s0168-9525(00)02024-2 10827456

[B30] Ronquist F. Teslenko M. van der Mark P. Ayres D. L. Darling A. Höhna S. (2012). MrBayes 3.2: Efficient Bayesian Phylogenetic Inference and Model Choice across a Large Model Space. Syst. Biol. 61, 539–542. 10.1093/sysbio/sys029 22357727 PMC3329765

[B31] Rozas J. Ferrer-Mata A. Sánchez-DelBarrio J. C. Guirao-Rico S. Librado P. Ramos-Onsins S. E. (2017). DnaSP 6: DNA Sequence Polymorphism Analysis of Large Data Sets. Mol. Biol. Evol. 34, 3299–3302. 10.1093/molbev/msx248 29029172

[B32] Sharp P. M. Li W.-H. (1987). The Codon Adaptation index-a Measure of Directional Synonymous Codon Usage Bias, and its Potential Applications. Nucl. Acids Res. 15, 1281–1295. 10.1093/nar/15.3.1281 3547335 PMC340524

[B33] Šmarda P. Bureš P. Horová L. Leitch I. J. Mucina L. Pacini E. (2014). Ecological and Evolutionary Significance of Genomic GC Content Diversity in Monocots. Proc. Natl. Acad. Sci. U.S.A. 111, E4096–E4102. 10.1073/pnas.1321152111 25225383 PMC4191780

[B34] Sueoka N. (1988). Directional Mutation Pressure and Neutral Molecular Evolution. Proc. Natl. Acad. Sci. 85, 2653–2657. 10.1073/pnas.85.8.2653 3357886 PMC280056

[B35] Tillich M. Lehwark P. Pellizzer T. Ulbricht-Jones E. S. Fischer A. Bock R. (2017). GeSeq - Versatile and Accurate Annotation of Organelle Genomes. Nucleic Acids Res. 45, W6–W11. 10.1093/nar/gkx391 28486635 PMC5570176

[B41] Wicke S. Schneeweiss G. M. de Pamphilis C. W. Müller K. F. Quandt D. (2011). The Evolution of the Plastid Chromosome in Land Plants: Gene Content, Gene Order, Gene Function. Plant Mol. Biol. 76, 273–297. 10.1007/sl1103-011-9762-4 21424877 PMC3104136

[B36] Wright F. (1990). The ‘effective Number of Codons' Used in a Gene. Gene 87, 23–29. 10.1016/0378-1119(90)90494-9 2110097

[B37] Wu L. Nie L. Xu Z. Li P. Wang Y. He C. (2020). Comparative and Phylogenetic Analysis of the Complete Chloroplast Genomes of Three *Paeonia* Section Moutan Species (Paeoniaceae). Front. Genet. 11, 980. 10.3389/fgene.2020.00980 33193580 PMC7533573

[B38] Xie J. Qian K. Si J. Xiao L. Ci D. Zhang D. (2018). Conserved Noncoding Sequences Conserve Biological Networks and Influence Genome Evolution. Heredity 120, 437–451. 10.1038/s41437-018-0055-4 29396421 PMC5889393

[B39] Xu Z. H. Jiang H. Ye D. P. Liu E. D. (2010). The Wild Orchids in Yunnan. Kunming: Yunnan publishing group corporation & Yunnan Science and Technology press.

[B40] Zheng G. Wei L. Ma L. Wu Z. Gu C. Chen K. (2020). Comparative Analyses of Chloroplast Genomes from 13 *Lagerstroemia* (Lythraceae) Species: Identification of Highly Divergent Regions and Inference of Phylogenetic Relationships. Plant Mol. Biol. 102, 659–676. 10.1007/s11103-020-00972-6 31997112

