# Supplementary figures and images for: Comparative Chloroplast Genomics and Phylogenetic Analysis of Thuniopsis and Closely Related Genera within Coelogyninae (Orchidaceae)
Source: Front Genet. 2022 Mar 24;13:850201. doi: 10.3389/fgene.2022.850201 (PMC8987740; doi:10.3389/fgene.2022.850201)

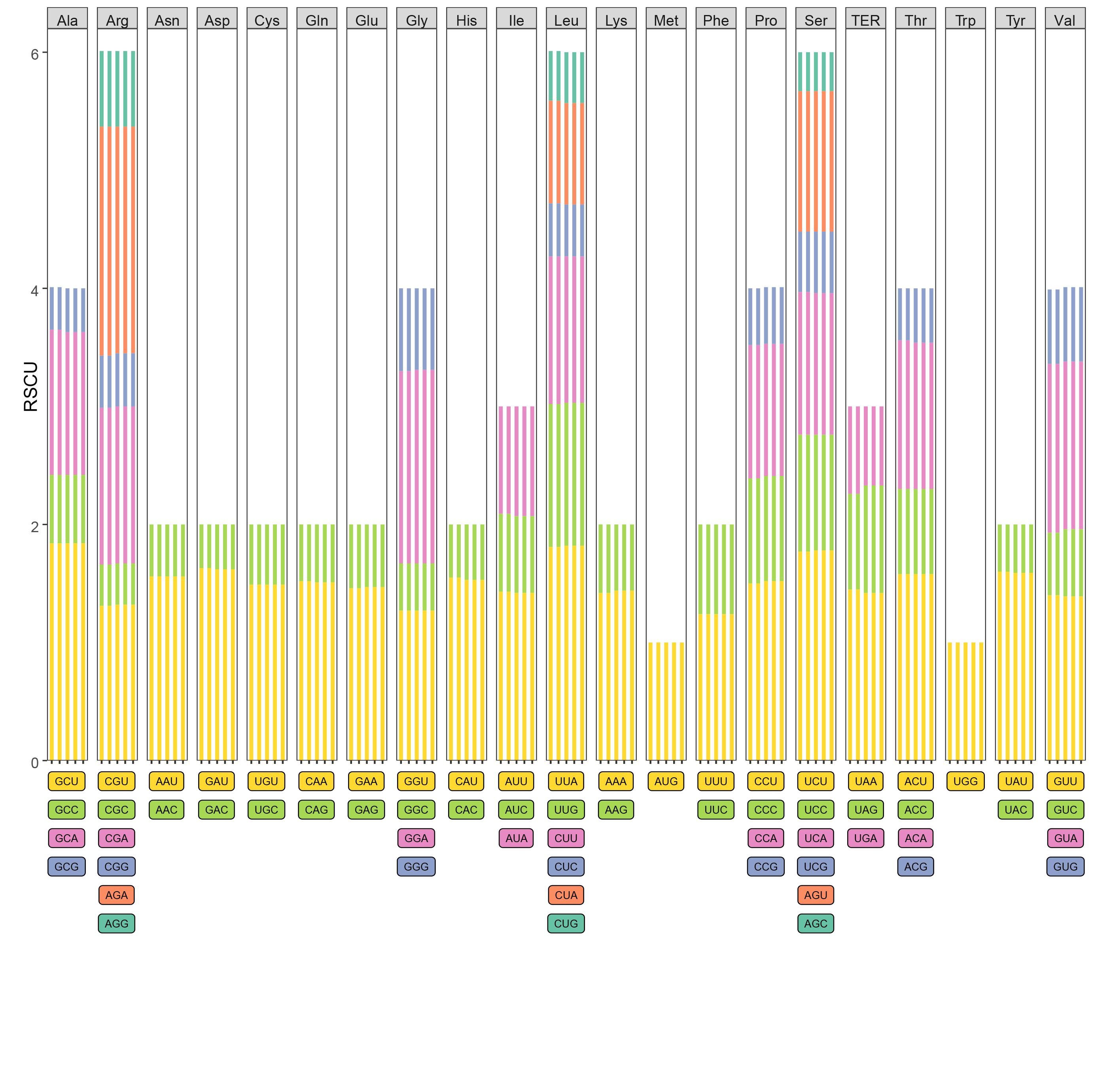

Supplement: Supplementary file 4 [file Image1.JPEG]
